# Adaptation to Fluconazole via Aneuploidy Enables Cross-Adaptation to Amphotericin B and Flucytosine in Cryptococcus neoformans

**DOI:** 10.1128/Spectrum.00723-21

**Published:** 2021-09-29

**Authors:** Feng Yang, Vladimir Gritsenko, Hui Lu, Cheng Zhen, Lu Gao, Judith Berman, Yuan-ying Jiang

**Affiliations:** a Department of Pharmacy, Shanghai Tenth People’s Hospital, School of Medicine, Tongji University, Shanghai, China; b Shmunis School of Biomedical and Cancer Research, The George S. Wise Faculty of Life Sciences, Tel Aviv University, Tel Aviv, Israel; Institut Pasteur

**Keywords:** amphotericin B, aneuploidy, cross-adaptation, *Cryptococcus neoformans*, fluconazole, flucytosine

## Abstract

The high morbidity and mortality of cryptococcal meningitis is due to the limited range of therapeutic options: only three classes of antifungal drugs are available (polyenes [amphotericin B], azoles [fluconazole], and pyrimidine analogues [flucytosine]). Fluconazole is the most widely used antifungal drug in sub-Saharan Africa, where cryptococcal meningitis is a major cause of death in patients infected with HIV. In this study, we found that exposure to fluconazole, even for short times (48 h) at subinhibitory concentrations, drove rapid adaptation of Cryptococcus neoformans serotype A strain H99 via the acquisition of different aneuploid chromosomes. These aneuploidies conferred heteroresistance to fluconazole. Importantly, most of the adaptors were cross-tolerant to flucytosine. Some of the aneuploid adaptors were not heteroresistant to fluconazole but were tolerant to amphotericin B. Thus, exposure to one antifungal drug class can promote adaptation to two antifungal drug classes, highlighting the plasticity of the C. neoformans genome and raising concerns about the rapid reduction in the range of treatment options for cryptococcal infections.

**IMPORTANCE** Cryptococcosis is a globally distributed invasive fungal infection caused by infections with Cryptococcus neoformans or Cryptococcus gattii. Only three classes of therapeutic drugs are clinically available for treating cryptococcosis: polyenes (amphotericin B), azoles (fluconazole), and pyrimidine analogues (flucytosine). Fluconazole is the primary drug available in resource-limited countries. Aneuploidy is a genomic state due to the gain or loss of chromosomes. We found that C. neoformans rapidly adapted to fluconazole by acquiring diverse aneuploidies and that specific aneuploidies enabled improved growth of isolates susceptible (tolerance) to amphotericin B and/or cross-tolerance to both fluconazole and flucytosine. Therefore, aneuploidy is an underlying mechanism of drug tolerance that not only arises rapidly during growth in fluconazole but can also confer tolerance to other antifungal drugs without prior exposure to those drugs. Resistant isolates have high MICs, and all cells grow similarly in medium with the drug, while tolerant isolates test as susceptible and grow slowly at drug concentrations above the MIC.

## INTRODUCTION

Cryptococcosis is a deadly opportunistic fungal infection primarily caused by Cryptococcus neoformans and Cryptococcus gattii. HIV infection is a major risk factor associated with the development of cryptococcosis and cryptococcal meningitis (CM), which cause 44% of HIV infection-related deaths in South Africa (1). Cryptococcal meningitis is diagnosed in approximately 90% of HIV-infected patients who have cryptococcosis (2, 3) and results in a mortality rate of about 70% in low-income countries (4) where access to antifungal drugs other than fluconazole is limited.

Antifungal treatment of cryptococcosis is limited to three classes of antifungal drugs: polyenes (amphotericin B), pyrimidine analogues (flucytosine), and azoles (fluconazole) (5). Amphotericin B (AMB) binds to fungal membrane ergosterol, causing changes in membrane permeability, leakage of ions, and cell death (6). Resistance to AMB is rare, from 0% to 5.8% for C. neoformans and C. gattii, respectively (reviewed in reference 6). Decreased membrane ergosterol content or changes in membrane sterol composition are the major causes of AMB resistance (6). Despite its renal toxicity, AMB remains the primary drug of choice for the treatment of cryptococcal infections in Western hospitals (7). However, because of the need to administer AMB intravenously and its high price, AMB is rarely used in Sub-Saharan Africa, where HIV-associated cryptococcal meningitis is prevalent.

Flucytosine (5FC) is a prodrug: it is imported by cytosine permease and converted to 5-fluorouracil (5FU) by cytosine deaminase, which is a fungus-specific enzyme. 5FU is further processed by uracil phosphoribosyl transferase, which inhibits both DNA and protein synthesis (8). 5FC resistance arises rapidly (7), via mutations either in genes encoding the cytosine permease or the uracil phosphoribosyl transferase or in *UXS1*, which encodes an enzyme that converts UDP-glucuronic acid to UDP-xylose for capsule biosynthesis (9). Thus, 5FC monotherapy is discouraged, but according to the new WHO guidelines in 2018, 1 week of 5FC and AMB or 2 weeks of 5FC and fluconazole are recommended as induction regimens for cryptococcal meningitis (10). Another report found that a combination of 5FC and fluconazole was more fungicidal against C. neoformans than fluconazole alone (11).

Fluconazole (FLC) is one of the most widely available and commonly used antifungal drugs. It is generally fungistatic, rather than fungicidal. FLC inhibits Erg11p, a cytochrome P450-dependent 14α-sterol demethylase that is essential for sterol biosynthesis. Inhibiting Erg11p prevents the conversion of lanosterol to ergosterol, depletes ergosterol, and causes the accumulation of toxic 14α-methyl sterols (12), which are synthesized via an Erg3p-dependent bypass pathway. Cryptococcal resistance to FLC can be caused by point mutations of *ERG11* (13, 14).

Sionov and coworkers studied the acquisition of FLC heteroresistance, a transient increase in strain MIC, in strain H99 and found a stepwise acquisition of aneuploidies due to sequential duplication of specific chromosomes (15, 16). H99 adapted to 32 μg/ml FLC via Chr1 disomy, and this strain also acquired Chr1 disomy plus Chr4 disomy at 64 μg/ml FLC. Further adaptation to 128 μg/ml FLC was achieved by disomy of additional chromosomes. However, the aneuploids were unstable. After daily passage in the absence of FLC, aneuploid chromosomes, as well as the resistance phenotype, were lost. This unstable resistance due to aneuploidy in C. neoformans is termed “heteroresistance” (16). *In vivo*, Chr1 disomy was frequently found in FLC-resistant colonies and was associated with clinical relapse during FLC monotherapy of HIV-associated cryptococcal meningitis patients (17). Chr1 harbors *ERG11*, which encodes the target of azole antifungals, as well as *AFR1*, which encodes an efflux pump that reduces intracellular FLC concentrations (16). Thus, in C. neoformans, as in Candida albicans, aneuploidy of one chromosome can mediate increased resistance by increasing the copy number of two genes that act additively (18, 19).

Here, we found that C. neoformans strain H99 simultaneously adapted to FLC via diverse aneuploidies when exposed to supra- or sub-MICs of FLC. Unexpectedly, some aneuploids were not heteroresistant to FLC, but rather, they became tolerant to AMB. Heteroresistant cells have high MICs and thus test as resistant to the drug, although the resistance phenotype is unstable; tolerance is defined as the ability of susceptible isolates to grow in the presence of supra-MIC drug levels. Other aneuploids became heteroresistant to FLC and tolerant to 5FC. Importantly, a short time of exposure to sub-MIC FLC concentrations was sufficient to select aneuploid adaptors that, depending upon the karyotype, were heteroresistant to FLC and tolerant to 5FC or FLC and hydrogen peroxide (H_2_O_2_). Thus, the plasticity of the C. neoformans genome challenges combinations of therapeutic strategies used to treat cryptococcosis.

## RESULTS

### Obtaining adaptors using inhibitory concentrations of fluconazole.

**FIG 1:**
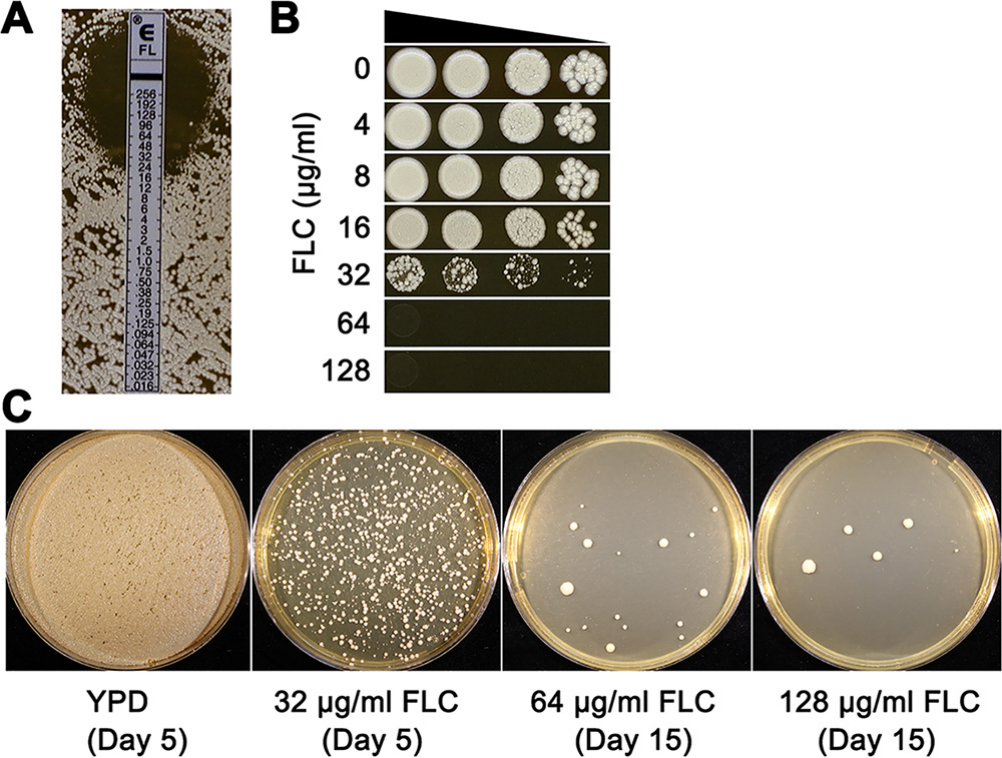
Obtaining adaptors from plates. (A and B) Susceptibility of parent strain H99 to fluconazole (FLC) was measured by Etest (A) and spot assay (B). For Etests, amounts of 1 × 10^5^ cells were spread on YPD plates. An Etest strip was applied to the center of the plate. In spot assays, cells were suspended in distilled water and serially diluted by 10-fold. Three microliters of each dilution was spotted on the plates containing the different drug concentrations as indicated in the figure. Plates were incubated at 30°C for 3 days and then photographed. (C) Approximately 1 × 10^6^ cells were spread on YPD plates supplemented with FLC concentrations as indicated in the figure. Plates were incubated at 30°C and photographed after 5 days (control and 32-μg/ml FLC plate) or 15 days (64-μg/ml and 128-μg/ml plates).

The FLC susceptibility of strain H99 was measured by Etest strips, and the MIC was 24 μg/ml (Fig. 1A). Spot assays detected obvious growth inhibition at 32 μg/ml of FLC. Interestingly, papillated growth was obvious at 32 μg/ml (Fig. 1B), suggesting that the ability to grow on supra-MIC FLC was a relatively high-frequency event. To test this hypothesis, approximately 1 × 10^6^ cells were spread on yeast extract-peptone-dextrose (YPD) plates supplemented with 32 μg/ml (YPD + 32 μg/ml FLC), 64 μg/ml, or 128 μg/ml of FLC. After 5 days of incubation on YPD + 32 μg/ml FLC at 30°C, approximately 1,054 colonies were obviously visible, indicating that the frequency of appearance of cells able to form colonies on YPD + 32 μg/ml FLC was approximately 0.1%. After 15 days, 16 and 5 adaptors, defined as colonies that grew on the selection plates, were obviously visible on YPD plates supplemented with 64 μg/ml and 128 μg/ml FLC, respectively (Fig. 1C). We randomly chose 27 adaptor colonies from YPD + 32 μg/ml and selected all the visible adaptors from the plates with 64 μg/ml and 128 μg/ml of FLC. Some adaptors grew poorly and were excluded from further analysis, yielding a total of 39 adaptors that were sent for whole-genome sequencing: 27 (P32-1 to P32-27), 9 (P64-1 to P64-9), and 3 (P128-1 to P128-3) colonies from YPD plates supplemented with 32 μg/ml, 64 μg/ml, and 128 μg/ml of FLC, respectively.

### Most fluconazole adaptors were aneuploid and acquired different levels of fluconazole heteroresistance.

**FIG 2:**
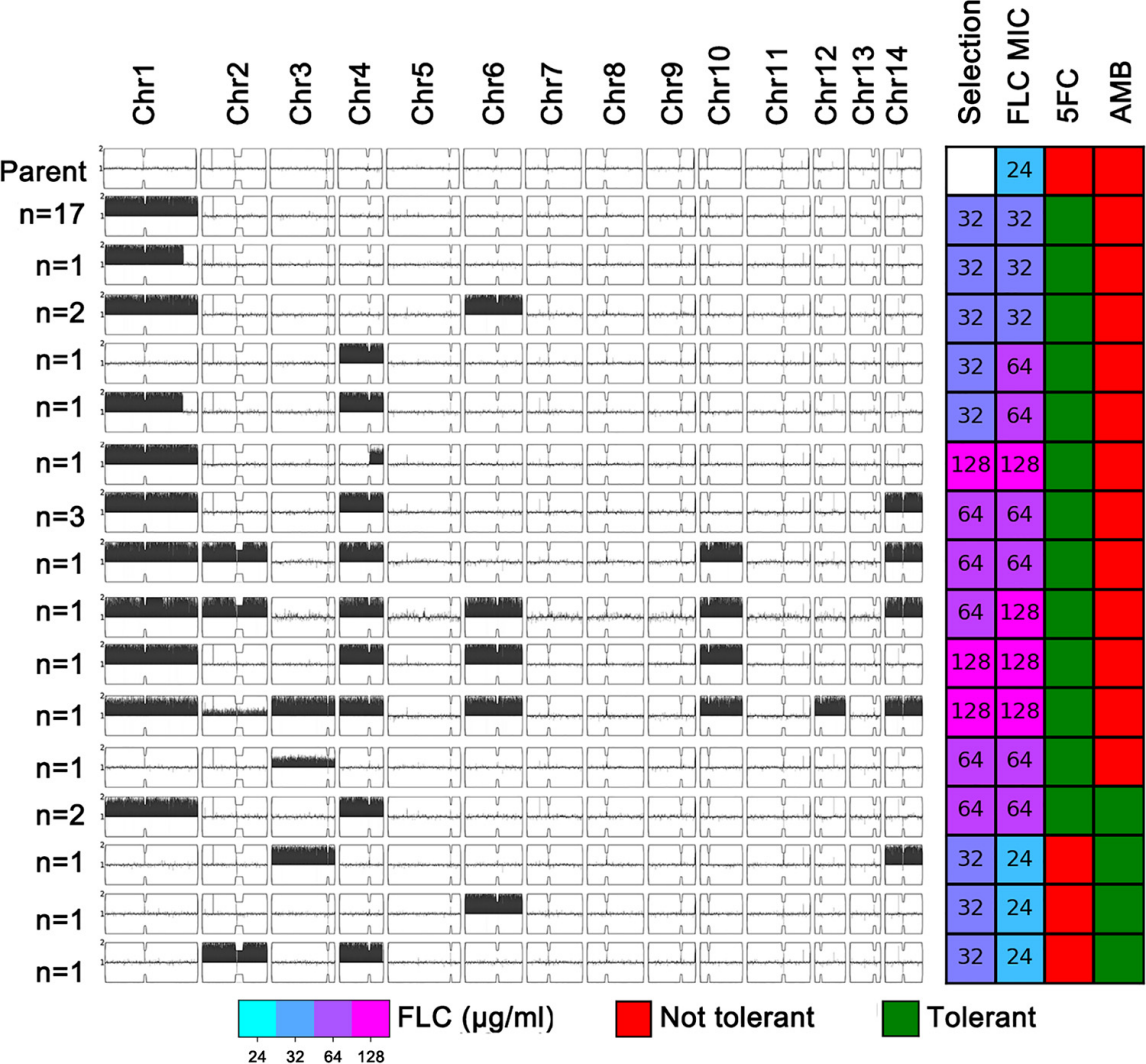
Karyotypic diversity and cross-tolerance profiles of fluconazole adaptors. Karyotypes of parent strain H99 and the 16 unique karyotypes for the 36 aneuploid isolates were visualized using YMAP (20). The chromosome copy number is indicated as a log_2_ ratio relative to that of the haploid H99 reference strain on the *y* axis, with 1 copy at the midline, clipped to show a maximum of 2 copies. The *x* axis shows the positions of the reads on each chromosome, mapped relative to the chromosome of reference strain H99, and the *y* axis shows the numbers of adaptors bearing the same karyotypes. The concentrations of FLCs from which isolates were derived (Selection) and the FLC MICs of the adaptors are indicated in the grid to the right, along with whether these karyotypes were tolerant or not tolerant to 5FC or AMB.

Analysis of the genome sequences of all 39 adaptors using YMAP (Yeast Mapping Analysis Pipeline) (20) revealed that only three of them (<8%) (P32-14, P32-19, and P64-5) were euploid. The remaining 36 adaptors were aneuploid (Fig. S1 in the supplemental material). Twenty of the aneuploids were aneuploid for a single chromosome. Among these, 17 adaptors were disomic for chromosome 1 (Chr1), and 1 adaptor had segmental disomy of Chr1. Sixteen of the 36 aneuploid adaptors were aneuploid for more than one chromosome. The most-frequent aneuploidy was Chr1 disomy: in total, 30 (>83% of the aneuploids) had either an extra copy of the whole chromosome 1 (28 adaptors) or a duplication of a large Chr1 segment (∼1.92 Mb) (2 adaptors). Of the 30 adaptors with an extra copy of most or all of Chr1, 12 (40.0%) also had other aneuploid chromosomes. The second-most-frequent aneuploidy (13 adaptors) was chromosome 4 disomy, but this was usually in combination with disomy of other chromosomes, especially chromosome 1 (85% of Chr4 disomy adaptors). Although disomies of Chr1 and Chr4 were the predominant chromosome changes, there was considerable diversity, with 16 unique karyotypes among the 36 aneuploids (Fig. 2). Furthermore, none of the adaptors had extra copies of chromosome 5, 7, 8, 9, 11, or 13.

Spot assays were performed to compare the adaptors to their parent for the ability to grow in the presence of FLC. All adaptors were tested on YPD plates supplemented with 32 μg/ml, 64 μg/ml, and 128 μg/ml of FLC, respectively. In addition to karyotype diversity, colonies derived from the same YPD + FLC plate also had diverse levels of ability to growth in the presence of FLC (Fig. 2 and Fig. S2). Among the 27 adaptors from 32 μg/ml FLC, most (21) adaptors grew at 32 μg/ml of FLC and 2 adaptors (P32-1 and P32-5) grew at 64 μg/ml FLC. P32-1 had Chr4 disomy, and P32-5 had Chr4 disomy plus segmental disomy of Chr1 (∼1.92 Mb). Two euploid adaptors (P32-14 and P32-19) and three aneuploid adaptors (P32-3, P32-21, and P32-22) failed to grow at 32 μg/ml FLC. P32-3 had Chr6 disomy. P32-21 had Chr2 disomy plus Chr4 disomy. P32-22 had Chr3 disomy plus Chr14 disomy. Thus, either these colonies were not *bona fide* adaptors or they had acquired a highly unstable mechanism of heteroresistance. Eight of the nine colonies from 64 μg/ml FLC were able to regrow on 64 μg/ml fluconazole, and all of them were aneuploid. One adaptor (P64-9) could grow at 128 μg/ml FLC and had multiple aneuploidies, including disomy of chromosomes 1, 2, 4, 6, 10, and 14. Each of the 3 adaptors derived from 128 μg/ml FLC had a unique karyotype, and they could regrow at 128 μg/ml FLC.

**FIG 3:**
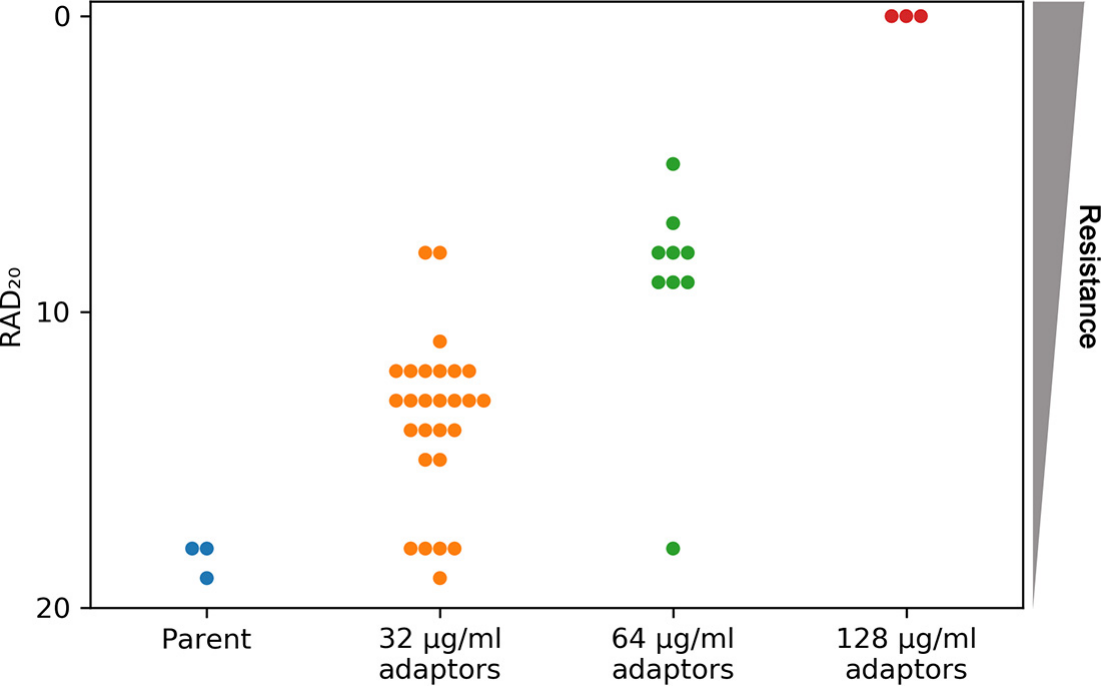
FLC susceptibility levels of adaptor colonies. FLC adaptors were tested using disk diffusion assays performed on YPD plates and disks containing 200 μg FLC. The RAD_20_, a reflection of the MIC, was calculated using *diskImageR* (22). Three individual colonies of the parent strain were tested, and all three values were plotted. For each of the adaptors, one colony was tested.

To ask if the ability to grow on YPD + FLC plates was due to heteroresistance, we performed disk diffusion assays (DDAs) using YPD plates with disks containing 200 μg FLC. Susceptibility was analyzed as the radius of the zone of inhibition (RAD) and was calculated using *diskImageR*. The RAD20, which is determined at the point where growth is reduced by 20%, was used as a parameter of heteroresistance (22). The adaptors that failed to grow at 32 μg/ml FLC in spot assays had susceptibilities similar to that of the parent strain. The other adaptors all had reduced RAD_20_s compared to that of the parent, indicating that they had become heteroresistant. The parent had a RAD_20_ of 18.3 ± 0.57. Adaptors that could grow at 32 μg/ml but not at 64 μg/ml FLC had RAD_20_ values of 11 to 15, adaptors that could grow at 64 μg/ml but not at 128 μg/ml FLC had RAD_20_ values of 7 to 9, and adaptors that could grow at 128 μg/ml had RAD_20_ values of 0 to 5 (Fig. 3).

Taken together, 92.3% (36 out of 39) of the adaptors were aneuploid. Disomy of Chr1 alone or in combination with disomy of other chromosomes was the most-prevalent event. Nonetheless, the adaptors had highly diverse karyotypes and different levels of heteroresistance to FLC. None of the euploid adaptors was heteroresistant to FLC. Some aneuploid adaptors also were not heteroresistant to FLC.

### Effect of short-time exposure to sub-MIC of fluconazole on adaptation.

**FIG 4:**
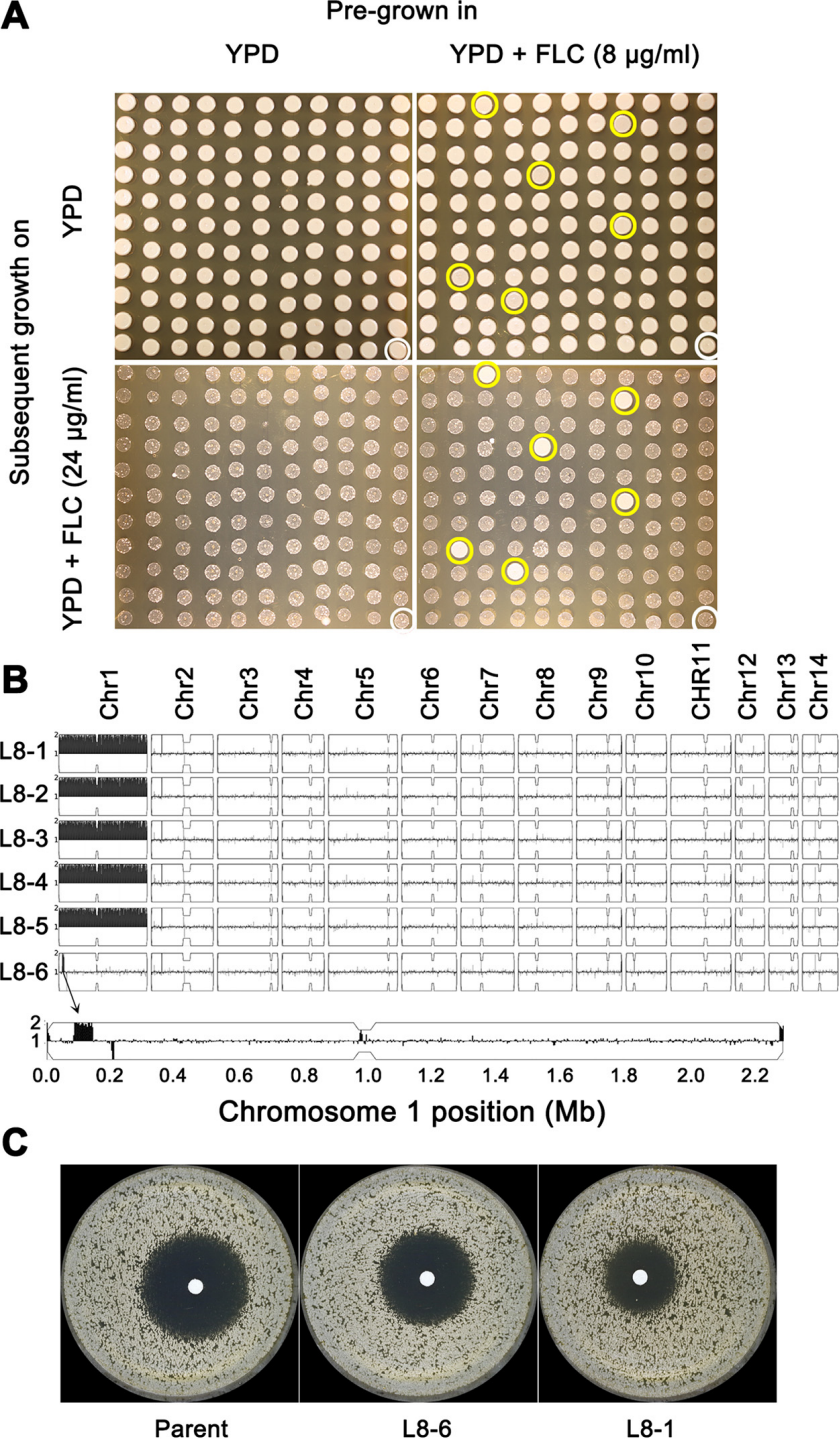
Adaptors from 48 h of exposure to sub-MIC fluconazole concentration. (A) H99 was grown in YPD or YPD supplemented with 8 μg/ml fluconazole (FLC) for 48 h. The culture was washed and diluted with water. Approximately 200 colonies were spread on YPD plates. From each plate, 120 colonies were randomly selected, along with a parent colony (white circles), and then tested on spot assays for tolerance to FLC. The plates were incubated at 30°C for 3 days and then photographed. Six colonies (yellow circles) were more tolerant than the parent. (B) The tolerant colonies (L8-1 to L8-6) were sequenced, and the karyotypes of the whole genomes, as well as of Chr1 for L8-6, were visualized using YMAP. (C) Disk diffusion assays with 200-μg-FLC disks of the parent, L8-6, and L8-1 are also shown.

H99, the parent strain, had an FLC MIC of 24 μg/ml (Fig. 1A), and growth was not inhibited at 8 μg/ml FLC (Fig. 1B). To test the effect of short-time exposure to sub-MICs of FLC on drug tolerance, H99 was grown in YPD broth supplemented with 8 μg/ml FLC for 48 h. Then, the culture was washed, diluted, and plated on YPD. After 72 h, 120 random colonies (putative adaptors) were isolated. Six adaptors (L8-1 to L8-6) grew at 24 μg/ml FLC, while the parent did not (Fig. 4A): five were disomic for Chr2 (L8-1 to L8-5). One of them (L8-6) had a segmental duplication of a small region (∼62 kb) of Chr1 that includes *ERG11*, which encodes the target protein of FLC (Fig. 4B), but does not include *AFR1*, which encodes an efflux pump. DDAs indicated that the parent had a RAD_20_ of 18.3 ± 0.57, that segmental disomy of Chr1 resulted in a slight increase in resistance (reduced radius of 15.3 ± 0.57), and that disomy of Chr1 caused a more obviously reduced radius (12.7 ± 0.57). Thus, whole-chromosome disomy of Chr1 conferred a higher level of heteroresistance than segmental disomy of Chr1 (Fig. 4C).

### Aneuploidy enables cross-tolerance to antifungal drugs.

**FIG 5:**
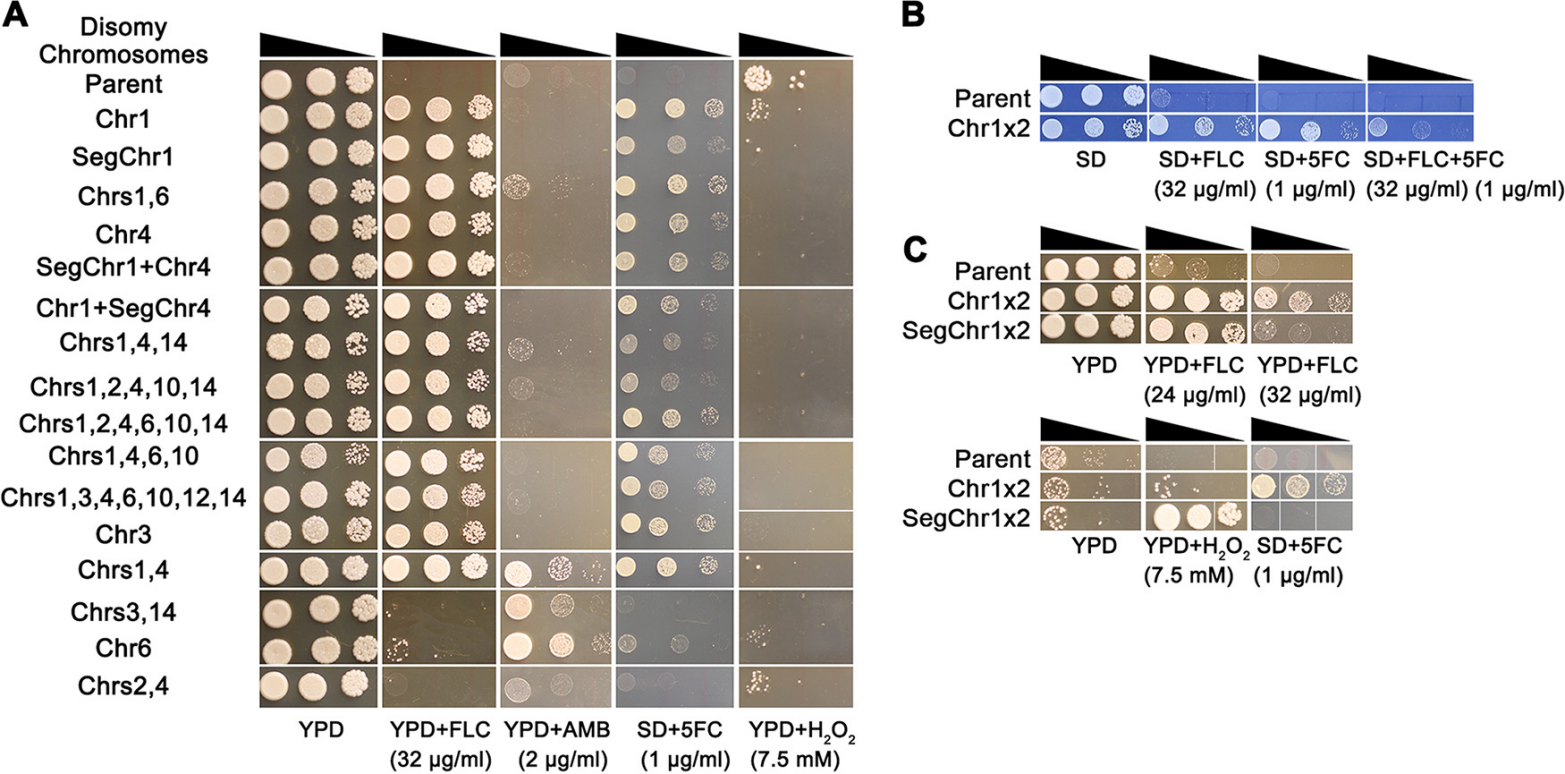
Cross-tolerance of aneuploids to antifungals. Fluconazole (FLC) adaptors obtained from YPD plates + FLC (≥32 μg/ml) (A) or YPD broth + FLC (8 μg/ml) (B and C) were tested for growth in FLC, amphotericin B (AMB), flucytosine (5FC), and hydrogen peroxide (H_2_O_2_). Adaptors representing unique karyotypes were tested. Cell were adjusted to 1 × 10^7^ cells/ml. Serial 10-fold dilutions of cell suspension was spotted (3 μl/spot) on the plates. The plates were incubated at 30°C for 3 days and then photographed. Tests of 5FC and the combination of 5FC and FLC were determined on SD plates; other drugs were tested on YPD plates.

We also tested the growth of the FLC adaptors on other antifungal drug classes and found that several of them were cross-tolerant to AMB or 5FC. We used 16 isolates, representative of the 16 unique aneuploid karyotypes obtained from YPD + FLC plates (Fig. 2). Two of the three karyotypes that did not cause heteroresistance to FLC were all tolerant to AMB. Even more surprisingly, all 13 karyotypes that retained the FLC heteroresistance were also tolerant to 5FC (Fig. 5A).

Importantly, one of the adaptors with both Chr1 disomy and Chr4 disomy was also cross-tolerant to AMB. Therefore, it was multidrug tolerant and could grow in the presence of drugs of all three classes of antifungal drugs used to treat cryptococcosis (Fig. 5A). However, isolates with segmental disomy of Chr1 plus disomy of Chr4, isolates with Chr1 disomy plus segmental disomy of Chr4, and isolates with Chr1 disomy plus Chr4 disomy plus additional aneuploidies were not multidrug tolerant (Fig. 2 and 5A); therefore, multidrug tolerance is a consequence of this specific double aneuploidy.

Two isolates obtained from a 48-h exposure to a sub-MIC of FLC (L8-1 [whole-chromosome disomy of Chr1] and L8-6 [segmental disomy of Chr1]) exhibited properties like those of Chr1 disomy adaptors obtained from YPD + FLC plates. L8-1 was cross-tolerant to FLC and 5FC and was able to grow in the presence of both drugs simultaneously (Fig. 5A and B). The segmentally disomic L8-6 was tolerant to FLC but not tolerant to 5FC; rather, it was cross-tolerant to hydrogen peroxide (H_2_O_2_) (Fig. 5C). None of the other aneuploid adaptors was cross-tolerant to H_2_O_2_ (Fig. 5A). Therefore, it appears that imbalances of different regions of Chr1 caused tolerance to FLC, 5FC, and/or H_2_O_2_.

## DISCUSSION

By isolating FLC adaptors following direct exposure to supra- and sub-MICs of FLC, we detected rapid adaptation within a single cycle of drug exposure. Furthermore, independent adaptors derived from one FLC concentration acquired diverse aneuploid karyotypes, with multiple isolates that had the same karyotype exhibiting very similar phenotypes with respect to FLC heteroresistance and other drugs. Interestingly, over 90% of the adaptors were aneuploid, and the majority carried Chr1 disomy and/or Chr4 disomy, irrespective of the FLC concentration used for selection. Importantly, most of the tested aneuploid colonies that had been selected on FLC were also cross-adapted to 5FC and/or AMB, without prior exposure to either 5FC or AMB. This raises a serious concern, given that exposure to the most widely used antifungal drug, FLC, rapidly yielded isolates tolerant to at least two and even all three of the antifungal drug classes available to treat cryptococcal meningitis.

The rapid adaptation to FLC and its association with diverse aneuploid karyotypes may be explained by the types of mitotic defects incurred when either C. albicans or C. neoformans is exposed to FLC. Specifically, a subpopulation of FLC-exposed cells undergoes a transient arrest in cell cycle progression that includes defective cytokinesis. This, in turn, yields tetraploid cells with chromosome instability that subsequently undergo high levels of chromosome loss, resulting in high levels of aneuploidy (21, 23). An alternative possibility is that aneuploids are formed directly upon exposure to FLC (24).

Karyotypic diversity arises from C. neoformans polyploid titan cells exposed to FLC as well. The genome sequences of four progeny of a single titan cell revealed one with Chr1 disomy, one with 2 copies of the right arm of Chr1, one with Chr4 disomy and Chr6 disomy, and one with Chr4 disomy and Chr10 disomy (25). These polyploid titan cells are intrinsically unstable, rapidly returning to haploidy in the absence of stress (25). Whether this mechanism is similar to or distinct from the mechanism of FLC-induced aneuploidy in haploid cells remains to be determined.

Almost all aneuploid adaptors (33 out of 36) were heteroresistant to FLC, with specific karyotypes causing higher heteroresistance. Colonies selected from 32 μg/ml FLC mostly (18 out of 27) had Chr1 disomy alone and a MIC of 32 μg/ml, colonies selected from 64 μg/ml FLC mostly (7 out of 8) had a MIC of 64 μg/ml, and colonies selected from 128 μg/ml FLC all had a MIC of 128 μg/ml. All three strains selected on 128 μg/ml FLC were disomic for Chr1 and Chr4 or a segment of Chr4, with two having additional chromosomes, including Chr6 and Chr10. These results are consistent with a previous report that sequential exposure of strain H99 to elevated concentrations of FLC resulted in a stepwise accumulation of disomies of different chromosomes (Chr1 disomy, then Chr1 disomy plus Chr4 disomy, and then additional Chr10 disomy with or without Chr14 disomy) (16). However, in this study, H99 was directly exposed to different concentrations of FLC until the first colonies appeared, with longer times required for colony appearance at higher FLC concentrations. This may be because the arrest of cell cycle progression in cells was dose dependent. Of note, some karyotypes caused FLC MICs higher than the selective conditions. For example, Chr4 disomy alone and Chr4 disomy plus segmental disomy of Chr1 appeared under 32-μg/ml FLC selection and enabled growth in up to 64 μg/ml of FLC, and one isolate disomic for Chr1, 2, -4, -5, -10, and -14 appeared under 64 μg/ml FLC selection and was able to grow in up to 128 μg/ml FLC. Therefore, instead of a stepwise increase of resistance, some aneuploid karyotypes enabled increased FLC tolerance in a single selective step.

Adaptors with the same karyotypes exhibited similar profiles of antifungal drug tolerance, which is consistent with the hypothesis that altered drug responses are due to aneuploidies in these strains. Nonetheless, not all disomic chromosomes have an additive effect on the ability to grow in FLC. For example, isolates with whole-Chr1 disomy or segmental disomy of 1.92 Mb of Chr1 had an FLC MIC of 32 μg/ml. Strains with Chr1 disomy plus Chr6 disomy retained the same MIC as strains with Chr1 disomy alone, and an isolate with Chr6 disomy alone had the same MIC as the parent. These results imply that Chr6 disomy does not affect the FLC MIC and that it is neutral, providing little fitness benefit or cost in FLC. Chr4 seemed to have different effects in different karyotypic contexts: when Chr4 disomy was the only aneuploidy, the strain had a MIC of 64 μg/ml. In addition, the following karyotypes that included Chr4 had the same MIC as when Chr4 disomy was the only aneuploidy: Chr1 disomy plus Chr4 disomy, segmental-Chr1 disomy plus Chr4 disomy, or Chr1 disomy plus Chr4 disomy plus Chr14 disomy. This suggests that Chr14 may also be neutral, with little or no benefit in FLC, However, Chr1 disomy plus segmental-Chr4 disomy had a higher MIC (128 μg/ml) than either Chr1 disomy or Chr4 disomy alone or disomy of both chromosomes, illustrating the potential for multiple genes on the same chromosome to modulate the dose-responsiveness of growth on FLC. Adaptors with more-disomic chromosomes usually had high MICs. For example, adaptors with disomy of 4, 6, or 7 chromosomes (Chr1, -4, -6, and -10; Chr1, -2, -4, -6, -10, and -14; or Chr1, -3, -4, -5, -10, -12, and -14, respectively) were able to grow in up to 128 μg/ml FLC.

Unexpectedly, three isolates selected on FLC did not grow better when retested on FLC. These included isolates with the following three karyotypes: Chr6 disomy alone, Chr2 disomy plus Chr4 disomy, and Chr3 disomy plus Chr14 disomy. One possible explanation for their selection and subsequent loss of the ability to grow in FLC is that they initially carried aneuploid chromosomes that conferred resistance and subsequently lost them when they were propagated without drug (between the selection and retesting experiment). Transient resistance (heteroresistance) to FLC due to unstable aneuploidy is well documented in C. neoformans (reviewed in reference 16). In Candida albicans, drug tolerance due to aneuploidy is also unstable in the absence of drug selection (26–28).

Importantly, all 13 karyotypes that caused heteroresistance to FLC were cross-tolerant to 5FC. Among them, 11 karyotypes were Chr1 disomic alone or in combination with at least one other disomic chromosome. In a recent study of H99, as well as other C. neoformans and C. gattii strains, 5FC selection caused Chr1 disomy that was attributed to *AFR1*, a gene on Chr1 that encodes an efflux pump. However, the effect of increased *AFR1* expression on 5FC tolerance was strain dependent (29). Taken together, these studies indicate that Chr1 disomy can be selected by growth on FLC or 5FC and that it confers cross-tolerance to both FLC and 5FC, irrespective of the drug used for the initial selection. This is reminiscent of Chr5 monosomy in C. albicans, which causes cross-tolerance to l-sorbose, caspofungin (fungicidal drug of the echinocandin family), and 5FC (26), and Chr2 trisomy, which causes cross-tolerance to caspofungin and hydroxyurea (a commonly used chemotherapeutic drug) (27). Thus, cross-tolerance to unrelated drugs via aneuploidy is a concern common to many pathogenic fungi and, in these studies, to both basidiomycete and ascomycete yeasts.

Of particular concern is the Chr1-plus-Chr4 disomy karyotype, which appeared twice among the isolates studied here. This karyotype conferred cross-tolerance to drugs from three classes of antifungals: FLC, AMB, and 5FC. Since these three drugs hail from the three major drug classes in clinical use, this type of multidrug tolerance has the potential to impede effective clinical treatment of cryptococcal infection. Importantly, the acquisition of two aneuploid chromosomes, whether in a single selection step or in a more gradual manner, has the potential to reduce the efficacy of the only available treatment options. Furthermore, given the use of AMB in induction therapy protocols, it will be important to investigate whether primary exposure to AMB gives rise to cross-tolerance to FLC and 5FC as well.

Aneuploid adaptors were selected after as little as 48 h of exposure to sub-MIC FLC concentrations (8 μg/ml). Among 120 random colonies, 5 had Chr1 disomy and one (segmental disomy of Chr1) was disomic for 62 kb of Chr1 that includes *ERG11* but not *AFR1.* Therefore, aneuploidy enabled rapid adaptation to both sub- and supra-MICs of FLC, and a short time of exposure to sub-MIC FLC was sufficient to select for aneuploidies that enabled tolerance to supra-MIC FLC. The heteroresistance in the segmental-Chr1 disomy was lower than for whole-Chr1-disomic aneuploids, probably because that segment includes only *ERG11* and not *AFR1*. Furthermore, duplication of whole Chr1 caused FLC and 5FC cross-tolerance, while duplication of one 62 kb region on Chr1 (adaptor L8-6) caused FLC and H_2_O_2_ cross-tolerance but not 5FC tolerance. Given that an extra copy of Erg11 on Chr1 is a major cause of FLC heteroresistance (16), this lack of 5FC tolerance in L8-6 implies that the ability to grow on 5FC resides elsewhere on Chr1, outside the 62 kb chromosomal segment. Whether the ability to survive H_2_O_2_ resides within the 62 kb retained in strain L8-6 remains to be determined.

In conclusion, C. neoformans strain H99 directly adapted to FLC at a range of concentrations. The adapted isolates had a range of karyotypes, with the most recurrent being Chr1 disomy, which conferred tolerance to FLC as well as to 5FC. Chr4 was the next-most-recurrent aneuploidy and, most importantly, two independent isolates with disomy of both Chr1 and 4 were able to grow in drugs from all major anticryptococcosis drug classes, raising concerns about the speed with which multidrug tolerance can arise in C. neoformans.

## MATERIALS AND METHODS

### Strains and culture conditions.

C. neoformans strain H99 was the parent strain used in this study. Stock cultures of all strains were preserved in 35% glycerol and maintained at −80°C. Cells were grown at 30°C in YPD medium (1% [wt/vol] yeast extract, 2% [wt/vol] peptone, and 2% [wt/vol] d-glucose) or synthetic defined (SD) agar plates (0.69% [wt/vol] yeast nitrogen base without amino acids, 2% [wt/vol] d-glucose, and 2% [wt/vol] agar).

### Etest assays.

H99 was streaked from −80°C stock onto YPD agar as described previously (27). After incubation at 30°C for 3 days, several colonies were chosen randomly and suspended in distilled water. Cell density was determined using a hemocytometer. Cells were adjusted to 1 × 10^6^ cells/ml. An amount of 100 μl of the culture was spread on YPD agar. An FLC Etest strip (bioMérieux, Marcy l’Etoile, France) was placed in the center of the plate. The plate was incubated at 30°C for 72 h and then photographed.

### Spot assays.

Cells were adjusted to 1 × 10^7^ cells/ml. Amounts of 3 μl of 10-fold serial dilutions was spotted on YPD agar plates supplemented with the compounds described in the figure legends. The plates were incubated at 30°C for 3 days and then photographed.

### Selection for fluconazole adaptors on agar medium.

H99 cells were adjusted to 1 × 10^7^ cells/ml. Amounts of 100 μl of cell suspensions were spread on YPD plates supplemented with FLC. The plates were incubated at 30°C for 5 days (control plate and 32-μg/ml FLC plate) or 15 days (64-μg/ml and 128-μg/ml FLC plates). Twenty-seven colonies (adaptors) from the 32-μg/ml FLC plate were randomly chosen, and all colonies that appeared on 64-μg/ml and 128-μg/ml FLC plates were also chosen for further analysis. The colonies were streaked on YPD plates (nonselective conditions) and incubated at 30°C for 3 days. For each adaptor, several colonies with similar sizes were collected, preserved in 35% glycerol, and maintained at −80°C.

### Selection of sub-MIC adapters by short-term-evolution YPD broth supplemented with fluconazole.

H99 cells (2.5 × 10^3^ cells/ml) were inoculated into YPD supplemented with 8 μg/ml FLC or without drug (control). After 48 h of growth at 30°C, the culture was washed and diluted with distilled water. Approximately 200 cells were spread on YPD plates. The plates were incubated at 30°C for 72 h. Randomly, 120 colonies from each plate were chosen for further analysis.

### Disk diffusion assays.

The CLSI M44-A2 guidelines (30) for antifungal disk diffusion susceptibility testing were followed with minor modifications. Strains were grown on YPD agar plates at 30°C, and cell density was adjusted to 1 × 10^6^ cells/ml. An amount of 100 μl of cell suspension was plated on each plate. On each petri plate, one filter disk (GE Healthcare, USA) supplemented with 200 μg FLC was placed in the center, and plates were incubated at 30°C and photographed at 72 h. Photographs were analyzed using the *diskImageR* pipeline (22).

### NGS.

Next-generation sequencing (NGS) was performed as described previously (28). To visualize the karyotypes, raw fastq files were uploaded to YMAP (version 1.0) (http://lovelace.cs.umn.edu/Ymap/) and read depth was plotted as a function of chromosome location using the reference genome of C. neoformans strain H99 (last modified 2015-04-01), as downloaded from NCBI (https://www.ncbi.nlm.nih.gov/assembly/GCF_000149245.1/). Chromosome end bias and GC content bias were corrected by YMAP as described in reference 20.

### Data availability.

The genome sequence data are available in the ArrayExpress database at EMBL-EBI (www.ebi.ac.uk/arrayexpress) under accession number E-MTAB-10177.
