# Supplementary material for: Adaptation to Fluconazole via Aneuploidy Enables Cross-Adaptation to Amphotericin B and Flucytosine in Cryptococcus neoformans
Source: Microbiol Spectr. 2021 Sep 29;9(2):e00723-21. doi: 10.1128/Spectrum.00723-21 (PMC8557924; doi:10.1128/Spectrum.00723-21)
Supplement: SUPPLEMENTAL FILE 1 — Supplemental material. Download SPECTRUM00723-21_Supp_1_seq2.pdf, PDF file, 6.4 MB [file spectrum00723-21_supp_1_seq2.pdf]

Supplemental materials

Figure S1. Karyotypes of all colonies selected on fluconazole

Karyotypes of parent strain H99 and all 39 sequenced colonies, (with their isolate number as indicated) were visualized using YMAP (1). The chromosome copy number is indicated as a $\log_2$ ratio relative to the haploid H99 reference strain on the y axis, with 1 copy at the midline, clipped to show a maximum of 2 copies. The x axis shows the positions of the reads on each chromosome, mapped relative to reference strain H99.

Figure S2. Spot assays detect growth in the presence of FLC

All 39 isolates were diluted in 10x steps from 10^7cells/ml to 10^5 cells/ml cells/ml (from high to low cell density as indicated by the black triangle) on YPD plates supplemented with FLC concentrations as indicated. Plates were incubated at 30˚C for 3 days and photographed.

Reference

1. Abbey DA, Funt J, Lurie-Weinberger MN, Thompson DA, Regev A, Myers CL, Berman J. 2014. YMAP: a pipeline for visualization of copy number variation and loss of heterozygosity in eukaryotic pathogens. Genome Med 6:100.

Figure S1

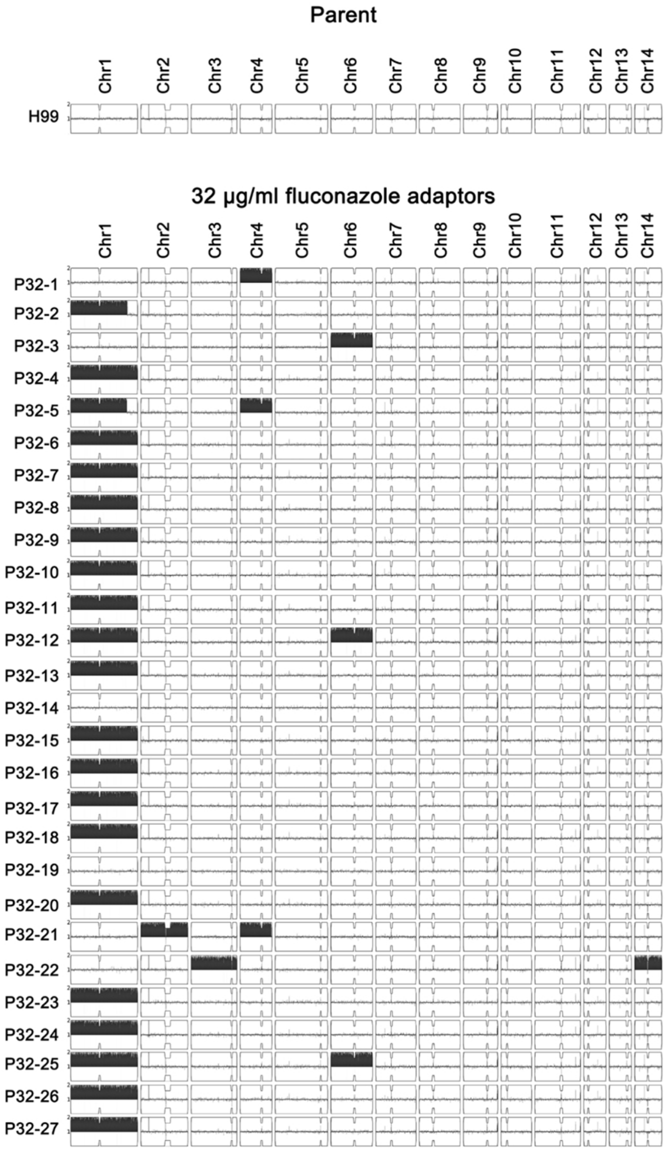
Parent
Chr1
Chr2
Chr3
Chr4
Chr5
Chr6
Chr7
Chr8
Chr9
Chr10
Chr11
Chr12
Chr13
Chr14
H99
32 µg/ml fluconazole adaptors
P32-1
P32-2
P32-3
P32-4
P32-5
P32-6
P32-7
P32-8
P32-9
P32-10
P32-11
P32-12
P32-13
P32-14
P32-15
P32-16
P32-17
P32-18
P32-19
P32-20
P32-21
P32-22
P32-23
P32-24
P32-25
P32-26
P32-27

64 μg/ml fluconazole adaptors

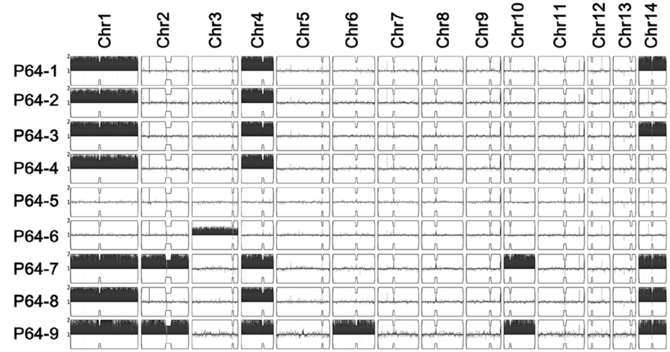

128 μg/ml fluconazole adaptors

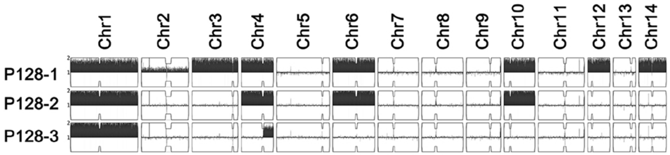

Figure S2

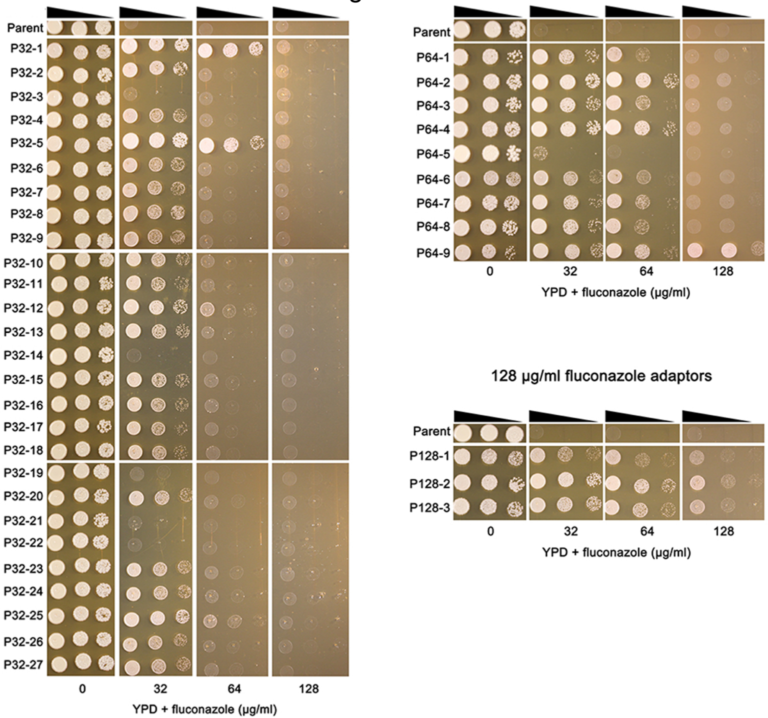
32 μg/ml fluconazole adaptors
Parent
P32-1
P32-2
P32-3
P32-4
P32-5
P32-6
P32-7
P32-8
P32-9
P32-10
P32-11
P32-12
P32-13
P32-14
P32-15
P32-16
P32-17
P32-18
P32-19
P32-20
P32-21
P32-22
P32-23
P32-24
P32-25
P32-26
P32-27
0
32
64
128
YPD + fluconazole (μg/ml)
64 μg/ml fluconazole adaptors
Parent
P64-1
P64-2
P64-3
P64-4
P64-5
P64-6
P64-7
P64-8
P64-9
0
32
64
128
YPD + fluconazole (μg/ml)
128 μg/ml fluconazole adaptors
Parent
P128-1
P128-2
P128-3
0
32
64
128
YPD + fluconazole (μg/ml)
